# Genome-wide association study reveals the advantaged genes regulating *japonica* rice grain shape traits in northern China

**DOI:** 10.7717/peerj.18746

**Published:** 2024-12-18

**Authors:** Hongwei Chen, Xue Zhang, Shujun Tian, Hong Gao, Jian Sun, Xiu Pang, Xiaowan Li, Quanying Li, Wenxiao Xie, Lili Wang, Chengwei Liang, Guomin Sui, Wenjing Zheng, Zuobin Ma

**Affiliations:** 1 Rice Research Institute of Liaoning Province, Liaoning Academy of Agricultural Sciences, Shenyang, China; 2 Rice Research Institute, Shenyang Agricultural University, Shenyang, China; 3 Liaoning Academy of Agricultural Sciences, Shenyang, China

**Keywords:** Japonica, Grain shape, GWAS, QTLs, Candidate genes

## Abstract

**Background:**

Rice, a staple food for over half of the global population, exhibits significant diversity in grain shape characteristics, which impact not only appearance and milling quality but also grain weight and yield. Identifying genes and loci underlying these traits is crucial for improving rice breeding programs. Previous studies have identified multiple quantitative trait loci (QTLs) and genes regulating grain length, width, and length-width ratio; however, further investigation is necessary to elucidate their regulatory pathways and their practical application in crop improvement.

**Methods:**

This study employed a genome-wide association study (GWAS) on 280 *japonica* rice varieties from northern China to decipher the genetic basis of grain shape traits. Phenotyping included measurements of 11 grain-related traits, such as grain length, width, and area, along with their brown and white rice counterparts. High-density single nucleotide polymorphism (SNP) markers (33,579) were utilized for genotyping, and GWAS was performed using a mixed linear model (MLM) incorporating principal component analysis (PCA) and kinship (K) matrix to account for population structure and relatedness.

**Results:**

Our analysis detected 15 QTLs associated with the 11 grain shape traits, of which five major QTL clusters emerged as crucial. Candidate genes, including *LOC_Os01g50720* (*qGL1*), *OsMKK4* (*LOC_Os02g54600*, influencing *qBA2*, *qWL2*, and *qWA2*), *GW5* (*LOC_Os05g09520*, controlling *qGW5*, *qBW5*, *qBR5*, *qWW5*, and *qWR5*), *GW6a* (*LOC_Os06g44100*, associated with *qGW6*, *qBW6*, *qBR6*, *qWW6*, and *qWR6*), and *FZP* (*LOC_Os07g47330*, linked to *qWL7*), were identified based on functional annotations and haplotype analysis. These findings offer valuable insights into the genetic mechanisms underlying rice grain shape and suggest promising targets for marker-assisted selection to enhance rice quality and yield.

## Introduction

The genetic mechanism of rice grain shape is a key field in the study of rice quality and yield (Li et al., 2022a). The characteristics that define rice grain shape, specifically grain length, grain width, and grain length-width ratio, not only play a pivotal role in the appearance and milling quality of rice but also serve as determinant factors influencing grain weight (Yang et al., 2021). Therefore, analyzing the genetic mechanism of rice grain shape, cloning the genes related to grain shape, and establishing the regulatory framework of grain shape formation provide important theoretical bases and gene resources for molecular breeding and high yields of rice, which are conducive to improving rice yield and quality (Xia et al., 2017). Based on the ratio of length to width in brown rice, rice grains can be categorized into short, medium, and long grains. The criteria for classification are as follows: grains with a ratio of ≥3.4 are considered long, those with a ratio of ≥2.3 but <3.4 are classified as medium, and grains with a ratio of <2.3 are designated as short (Awad-Allah et al., 2022). Among them, the long-grain varieties are becoming increasingly favored due to their aesthetically pleasing appearance and their reduced chalkiness in milled rice (Singh et al., 2017).

There are many ways to regulate rice grain shape, including ubiquitin proteases, hormones, epigenetic modifications, *etc*., which lead to the changes in grain shape through the change of cell number or cell volume (Meng et al., 2022). Many genes and QTLs controlling grain length have been cloned, such as *qGL3*, *SMOS1*, *GS3*, *GS2*, *PGL1*, *TGW6*, *PGL2*, *GL7* and others (Fan et al., 2006; Zhang et al., 2012; Ishimaru et al., 2013; Duan et al., 2014; Kabir & Nonhebel, 2021). Among them, *qGL3* encodes a protein phosphatase OsPPKL1 containing two Kelch functional domains, which plays the role of negative regulator in the regulation of rice grain length, and the Kelch functional domain is sufficient and necessary in the negative regulatory function of OsPPKL1 (Zhang et al., 2012; Gao et al., 2019). *SMOS1* encodes a transcription factor in rice, that includes an AP2 domain, which regulates grain size through the expansion of cell number and the regulation of cell size (Qiao et al., 2017; Hirano et al., 2017). *TGW6* encodes an IAA-glucose hydrolase, which influences the number of grain cells and the length of grain by regulating the supply of IAA (Ishimaru et al., 2013; Kabir & Nonhebel, 2021). *GL7* encodes a LONGIFOLIA protein, which is highly homologous to the LONGIFOLIA protein of *Arabidopsis thaliana*. The increase of *GL7* expression can increase the longitudinal cell division of grain and reduce the transverse cell division, resulting in an increase in grain length (Wang et al., 2015a, 2015b). *GS3*, located in the centromere region of chromosome 3, is a major gene controlling grain length (Fan et al., 2006; Kan et al., 2022). The G-protein-γ subunit encoded by *GS3* contains a plant-specific organ size regulation (OSR) domain, the deletion of which leads to increased grain length in rice (Mao et al., 2010). In addition, *GW2*, *GS5*, *GW5*, *GS6* and *GW8* were the major genes that had been cloned to affect grain width. Among them, *GW2* encodes a RING-type protein with E3 ubiquitin ligase activity, which can function in the ubiquitin-proteasome pathway to degrade proteins. The loss or decrease of *GW2* expression can lead to the increase of the number of rice glume cells, which in turn increases the grain width, the grain filling speed, the grain weight and the yield of rice (Song et al., 2007; Hao et al., 2021). *GW5* encodes a nuclear localization protein that inhibits the activity of glycogen synthase kinase GSK2, thereby regulating the expression level of brassinolide response genes and grain growth response (Weng et al., 2008; Liu et al., 2017). Through the study of *GW5* genes in different cultivated rice varieties, it was found that the grain width increased due to the deletion of gene fragments under the action of both artificial and natural selection in the process of rice domestication (Liu et al., 2017). *GS5* is a major gene on chromosome 5 that affects grain width, filling and grain weight, and positively regulates rice grain length by encoding serine carboxypeptidase (Li et al., 2011; Xu et al., 2015).

At present, QTL research on grain shape related traits of rice has been widely carried out, mainly to improve the appearance quality of rice, but only a few QTLs have been applied in current breeding programs. Further characterization of QTLs associated with rice grain shape and the cloning of the underlying genes is essential. Additionally, a deeper understanding of the regulatory pathways governed by these genes is necessary to fully exploit their potential in rice breeding programs aimed at improving grain shape traits. With the development of high-throughput sequencing technology, high density SNP markers covering the whole genome have made genome-wide association studies widely used. In the past 10 years, the genetic research of plant quantitative traits based on GWAS has made great progress. *GLW7* represents a pivotal rice grain type regulatory gene that has been successfully cloned through the application of GWAS (Si et al., 2016). Lv et al. (2019) conducted a genome-wide association analysis on grain shape-related traits, including grain length, grain width, thousand-grain mass, and length-to-width ratio, using 161 indica rice varieties along with 16,352 SNPs, and identified a total of 38 significant loci. Feng et al. (2016), on the other hand, utilized 469 rice varieties combined with 5,291 SNPs to detect 11 novel QTLs through genome-wide association analysis. Grain shape is not only one of the determining factors of yield traits, but also affects the appearance and milling quality of rice.

In this study, 280 *japonica* rice varieties from northern China were used to conduct genome-wide association study for grain-shape-related traits, and to explore the relevant loci or genes controlling grain shape. These results will provide a theoretical basis for elucidating the genetic mechanism of rice grain shape and breeding excellent new rice varieties with excellent grain shape.

## Materials and methods

### Plant materials

The association panel consisted of 280 *japonica* accessions from northern China (Table S1). These accessions were planted in the experimental field of Liaoning Rice Research Institute (41°N, 123°E) from April to October 2021. The sowing, transplanting and harvesting dates were April 25, May 20 and October 10, respectively. Each accession was planted in a plot of three rows, with eight plants in each row at a spacing of 16.7 × 30 cm, and this was replicated twice. Fertilizer for the cultivated land was 180 kg N ha^−1^, 60 kg P_2_O_5_ ha^−1^, and 120 kg K_2_O ha^−1^, with attention to weeding and pest control.

### Phenotyping

When the accessions were mature, five plants were selected in the middle of the plot for harvesting and placed in a cool place to allow the seeds dry naturally. A seed moisture meter is used to detect the grain moisture content, and about 200 grains were selected when it has been reduced to 12–14%. For brown rice, the outer husk is removed, retaining the bran layer and germ. To produce white rice, further milling removes the bran layer and germ using a CLS JNM-1 rice husker. Grain length (GL), grain width (GW), grain length-width ratio (GR), brown rice length (BL), brown rice width (BW), brown rice length-width ratio (BR), brown rice area (BA), white rice length (WL), white rice width (WW), white rice length-width ratio (WR) and white rice area (WA) are measured using a rice appearance quality detection analyzer (SC-E, Hangzhou Wanshen Test Technology Corporation, Hangzhou, China). Three technical repeats and two biological repeats were performed (average 120 seeds per plant), and the mean values were calculated for subsequent analysis.

### Genotyping

DNA was extracted using the SDS lysis method (Zhang et al., 2019). The leaves of tillering stage were frozen with liquid nitrogen and ground with tissue grinder. SDS lysis buffer was added and incubated at 65 °C for 30 min. Sodium acetate was then added, the mixture was thoroughly mixed, and centrifuged. Isopropyl alcohol was added to the supernatant to precipitate the DNA, which was then rinsed twice with 70% ethanol and air-dried. The genotypic data were obtained using a 40K liquid-phase sequence chip (Li et al., 2022c), and all raw reads were filtered for high quality, with Q20 quality scores >95% and guanine-cytosine (GC) content <50%. Finally, 33,579 high-quality SNPs were obtained and used for genome-wide association study (GWAS) in this study. The genotypic data described here are accessible *via* FigShare (https://doi.org/10.6084/m9.figshare.26461429.v1).

### Population genetic analysis

Principal component analysis (PCA) was conducted using the efficient mixed-model association (EMMA) method in the Genome Association and Prediction Integrated Tool (GAPIT) R package (Lipka et al., 2012) to examine the population structure. Kinship analysis of accessions is performed using the GAPIT package of R software and used for subsequent GWAS analysis. The K matrix (kinship matrix) was calculated by the EMMAX software (Kang et al., 2010) based on the Bayesian network (BN) method using those high-quality SNPs.

### GWAS analyses

We performed a GWAS to detect SNPs that were significantly associated with all grain-shape-related traits using high-quality SNPs and the mean trait values of the 280 accessions. Marker-trait associations were conducted by the mixed linear model (MLM), PCA+K, implemented in the GAPIT package in R software (version 4.3.1) (Lipka et al., 2012). The critical *P*-value for declaring significant marker-trait association (1.0 × 10^–4^) was calculated using GEC software based on the independent effective SNP number (Li et al., 2012). To estimate independently associated regions of identified QTLs, significantly trait-associated SNPs situated in one estimated LD block were defined as the same QTL. Each LD block containing the identified SNPs was evaluated using the R package “LDheatmap” (Shin et al., 2006; Yano et al., 2016).

### Identification of candidate genes

Haplotype analysis was conducted based on non-synonymous SNPs of QTL candidate genes in the temperate *japonica* rice of the 3K program (The 3,000 rice genomes project, 2014). Haplotypes containing more than 10 accessions were used to analyze significant differences in phenotype. Five representative candidate genes were selected for a comprehensive analysis based on the significance of the haplotype analyses (analysis of variance (ANOVA)), their biochemically related functions, and their expression profiles.

### Statistical analysis

Differences in mean phenotypic values between haplotypes (consisting of more than 10 germplasm) were assessed using one-way ANOVA. Duncan’s multiple means comparison test uses the agricolae package in R software (4.3.1) to determine the significance of any difference (5% significance level). Phenotypic correlation analysis of grain-shape-related traits was calculated using the corrplot package in R. The variance components were evaluated using multi-site analysis, and all effects were treated as random.

## Results

### Phenotypic variation of grain-shape-related traits

**Figure 1:**
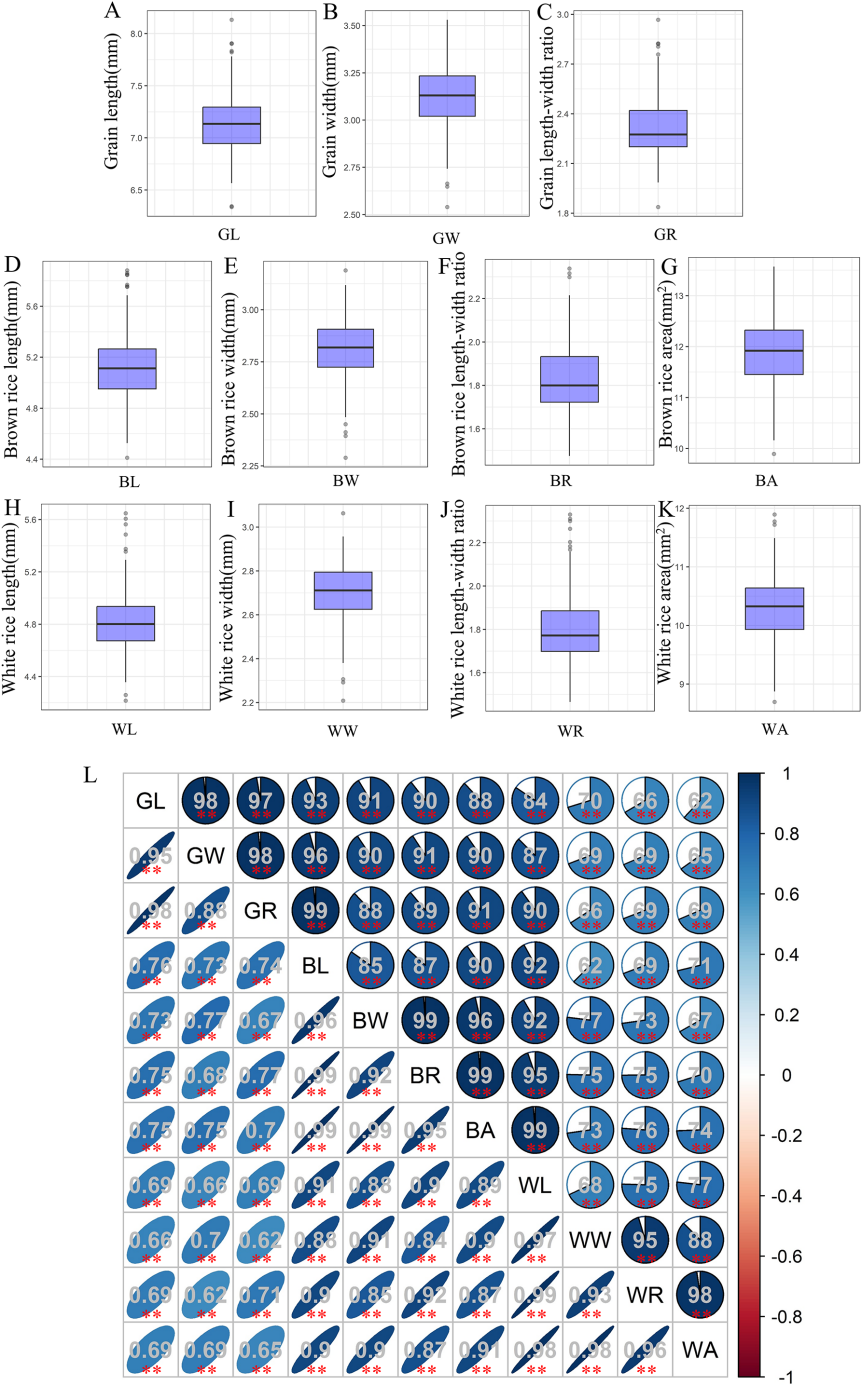
Values and correlations of grain-shape-related traits. (A–K) Box plots of grain length (A), grain width (B), grain length-width ratio (C), brown rice length (D), brown rice width (E), brown rice length-width ratio (F), brown rice area (G), white rice length (H), white rice width (I), white rice length-width ratio (J), and white rice area (K). GL, grain length; GW, grain width; GR, grain length-width ratio; BL, brown rice length; BW, brown rice width; BR, brown rice length-width ratio; BA, brown rice area; WL, white rice length; WW, white rice width; WR, white rice length-width ratio; WA, white rice area. (L) Correlations between the eleven tested traits. The areas and colors of ellipses correspond to the absolute values of the corresponding correlation coefficients. **Denotes significant correlations at *P* < 0.01.

The associations used in this study showed wide variations for grain-shape-related traits, and most traits appeared to be normally distributed (Table S1). Among them, the average GL, GW, and GR values of 280 accessions were 7.14, 3.12, and 2.32 mm, respectively (Figs. 1A–1C). Compared with grain, both brown rice and white rice exhibit decreases in both length and width to varying degrees. The mean values of BL, BW, and BR were 5.12, 2.81, and 1.83 mm, respectively (Figs. 1D–1F), while the average WL, WW, and WR values were only 4.84, 2.71, and 1.8 mm, respectively (Figs. 1H–1J). In addition, the average BA and WA values were 11.9 and 10.29, respectively (Figs. 1G, 1K). Correlation analysis showed that all 11 grain-shape-related traits were positively correlated, and the correlation coefficients between GR and BL, BW, and BR reached 0.99 (Fig. 1L).

### Phylogenetic and population structure analysis

**Figure 2:**
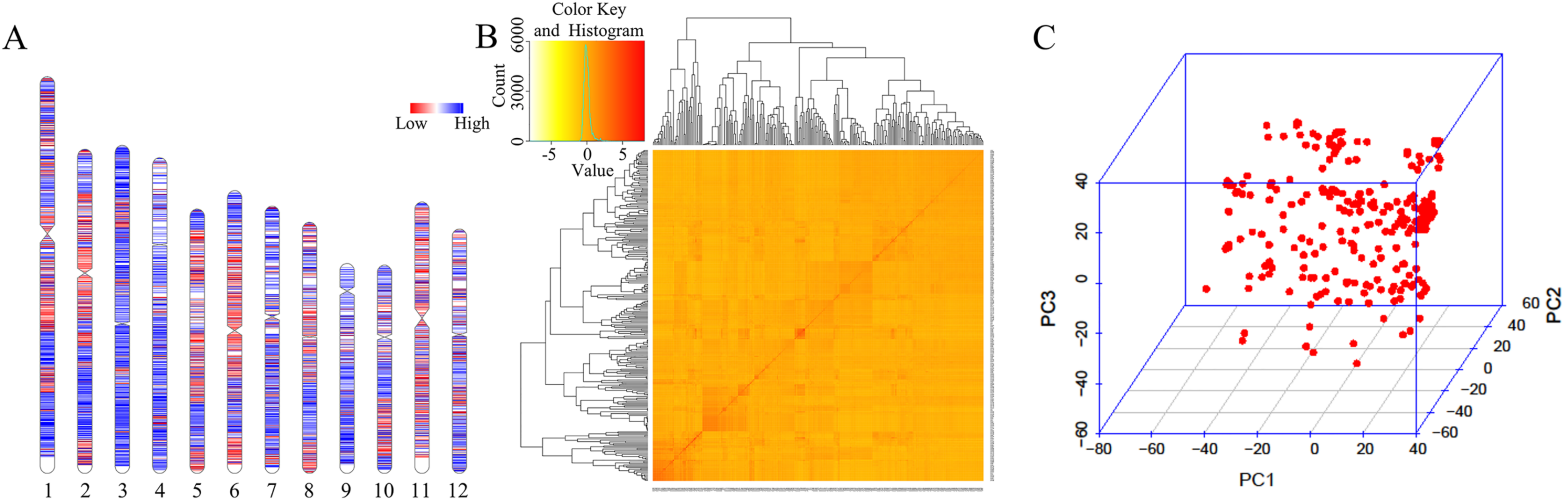
Genotypic analysis. (A) Heatmap illustrating SNP density in 280 accessions. (B) Heatmap of the kinship matrix, indicating the degree of relatedness between each pair of accession in the dataset. Darker colors indicate greater relatedness. (C) PCA of 280 accessions based on the screen plot within the rice diversity panel.

A total of 33,579 high-quality SNPs were obtained, and the average marker spacing was 11.1 kb. Among them, chromosome 1 had the most SNPs, containing 4,283, and the average marker spacing was 10.1 kb. Chromosome 9 contained the least number of markers, 1,986, with an average marker spacing of 11.5 kb (Fig. 2A, Table S2). Kinship and principal component analysis (PCA) were used to analyze the genetic structure of the SNPs in the 280 associations. The results showed that there were no obvious population structure groups among the 280 associations, because all of them came from northern China (Figs. 2B–2C).

### GWAS analysis of grain-shape-related traits

To identify genomic regions associated with the measured phenotypes, GWAS was performed on 280 associations. The Manhattan plot showed that when an association threshold of 1.0 × 10^−4^ was set, a total of 15 SNPs associated with grain shape were identified across the 280 associations, including one (*qGL1*), two (*qGW5* and *qGW6*), one (*qBA2*), two (*qBR5* and *qBR6*), two (*qBW5* and *qBW6*), one (*qWA2*), two (*qWR5* and *qWR6*), two (*qWL2* and *qWL7*), and two (*qWW5* and *qWW6*) for GL, GW, BA, BR, BW, WA, WR, WL, and WW, respectively (Table 1, Fig. S1). The identification of QTLs was achieved by defining contiguous SNPs exhibiting significant correlation and residing within an estimated linkage disequilibrium (LD) block. This approach yielded a total of five distinct QTL intervals for the 11 traits under study. Among these, *qGL1* was found to influence GL, while *qBA2* exhibited a multi-faceted effect, influencing BA, WL, and WA simultaneously. Additionally, two QTLs, *qGW5* and *qGW6* (despite being sequentially numbered, they both affect the same set of traits), were identified to have a combined influence on GW, BW, BR, WW, and WR. Lastly, a unique QTL, *qWL7*, was determined to specifically affect WL. These findings are summarized in Table 1.

**Table 1:** QTLs identified for grain-shape-related traits by GWAS.

Trait	QTL	Chr	Peak SNP	Interval (Mb)	*P*-value of peak SNPs	Previously reported QTLs and gene
GL	*qGL1*	1	rs1_29399705	29.07–29.41	9.00439E−05	
GW	*qGW5*	5	rs5_5400007	5.30–5.46	1.22E−08	*GW5* (Weng et al., 2008)
	*qGW6*	6	rs6_26455250	26.40–26.68	4.09872E−05	*GW6a* (Song et al., 2015)
BW	*qBW5*	5	rs5_5299885	5.30–5.46	2.98E−07	*GW5*
	*qBW6*	6	rs6_26455250	26.40–26.68	7.31E−05	*GW6a*
BR	*qBR5*	5	rs5_5460890	5.30–5.46	6.40E−09	*GW5*
	*qBR6*	6	rs6_26455250	26.40–26.68	6.13E−08	*GW6a*
BA	*qBA2*	2	rs2_33442570	33.04–33.50	2.27E−05	*OsMKK4* (Duan et al., 2014)
WL	*qWL2*	2	rs2_33357184	33.04–33.50	0.000037437	*OsMKK4*
	*qWL7*	7	rs7_28430999	28.17–28.64	1.70739E−05	*FZP* (Ren et al., 2018)
WW	*qWW5*	5	rs5_5460942	5.30–5.46	4.12117E−08	*GW5*
	*qWW6*	6	rs6_26455146	26.40–26.68	1.70995E−06	*GW6a*
WR	*qWR5*	5	rs5_5400006	5.30–5.46	2.59302E−09	*GW5*
	*qWR6*	6	rs6_26455250	26.40–26.68	1.36456E−10	*GW6a*
WA	*qWA2*	2	rs2_33442570	33.04–33.50	5.41E−06	*OsMKK4*

### Candidate gene identification

**Figure 3:**
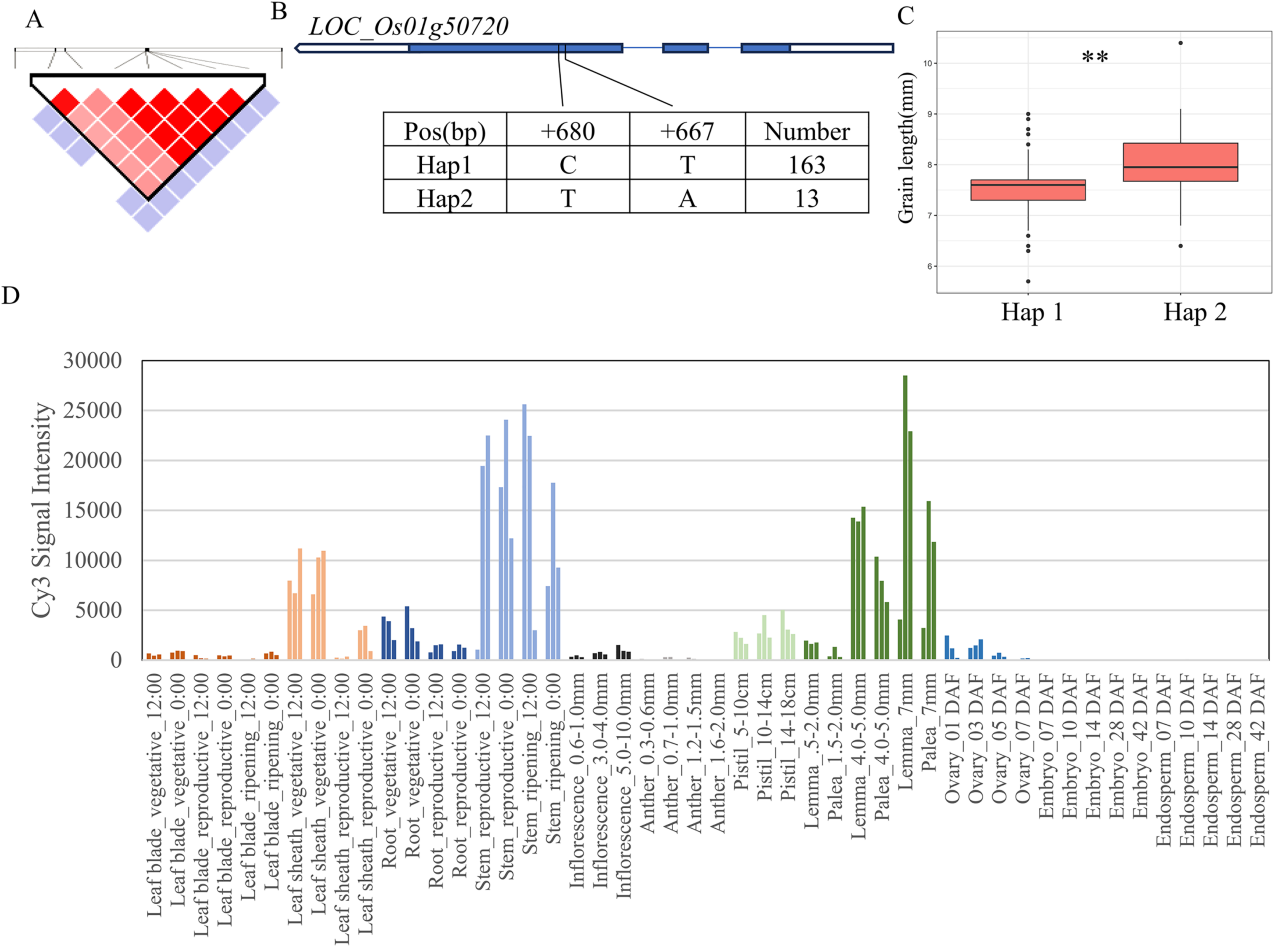
Candidate genes analysis of *qGL1* on chromosome 1. (A) Linkage disequilibrium (LD) block surrounding the peak on chromosome 1. (B) Gene structure of *LOC_Os01g50720* and DNA polymorphisms within that gene. Blue boxes, white boxes and straight lines represent exons, introns and untranslated regions (UTR), respectively, and arrows represent gene directions. (C) Boxplots illustrating grain length based on haplotypes for *LOC_Os01g50720* using non-synonymous single nucleotide polymorphisms (SNPs) within the coding region. **Denotes the significance of ANOVA at *P* < 0.01. (D) Spatio-temporal expression patterns of *LOC_Os01g50720* in various Nipponbare tissues throughout the entire growth period in the field (downloaded from RiceXPro (version 3.0)).

For the 11 grain-shape-related traits, we performed haplotype analysis to identify candidate genes for five QTLs consistently identified in five chromosomal regions (Table 2). Based on the Nipponbare reference genome IRGSP 1.0, the 29.07–29.41 Mb (336 kb) region containing *qGL1* on chromosome 1 (Fig. 3A) harbored 53 candidate genes, among which four genes had known annotations in the Rice Annotation Project Database (RAP-DB, https://rapdb.dna.affrc.go.jp/). *LOC_Os01g50720* encodes an OsMYB14 transcription factor and is a member of the rice MYB transcription factor gene family. The 3K program display that, a total of 2 SNPs were found in the coding region of *MYB14* (Fig. 3B), forming two haplotypes, among which the GL of Hap1 was significantly higher than that of Hap2 (Fig. 3C). According to the published rice gene expression profile database (RiceXPro (version 3.0)), *OsMYB14* is relatively highly expressed in specific organs (stem, lemma and palea) (Fig. 3D). Therefore, *LOC_Os01g50720* is one of the most likely candidate genes in this region.

**Table 2:** List of the five most likely candidate genes for five quantitative trait loci (QTLs) associated with grain-shape-related traits.

QTL	Candidate gene	MSU_Locus_Annotation	Known function
*qGL1*	*LOC_Os01g50720*	MYB family transcription factor, putative, expressed	
*qBA2, qWL2, qWA2*	*LOC_Os02g54600*(*OsMKK4*)	STE_MEK_ste7_MAP2K.5–STE kinases include homologs to sterile 7, sterile 11 and sterile 20 from yeast, expressed	Influences grain size in rice
*qGW5, qBW5, qBR5, qWW5, qWR5*	*LOC_Os05g09520*(*GW5*)	IQ calmodulin-binding motif family protein, expressed	Regulate grain width in rice
*qGW6, qBW6, qBR6, qWW6, qWR6*	*LOC_Os06g44100*(*GW6a*)	HLS, putative, expressed	Regulate grain width in rice
*qWL7*	*LOC_Os07g47330*(*FZP*)	AP2 domain containing protein, expressed	Influences grain size in rice

**Figure 4:**
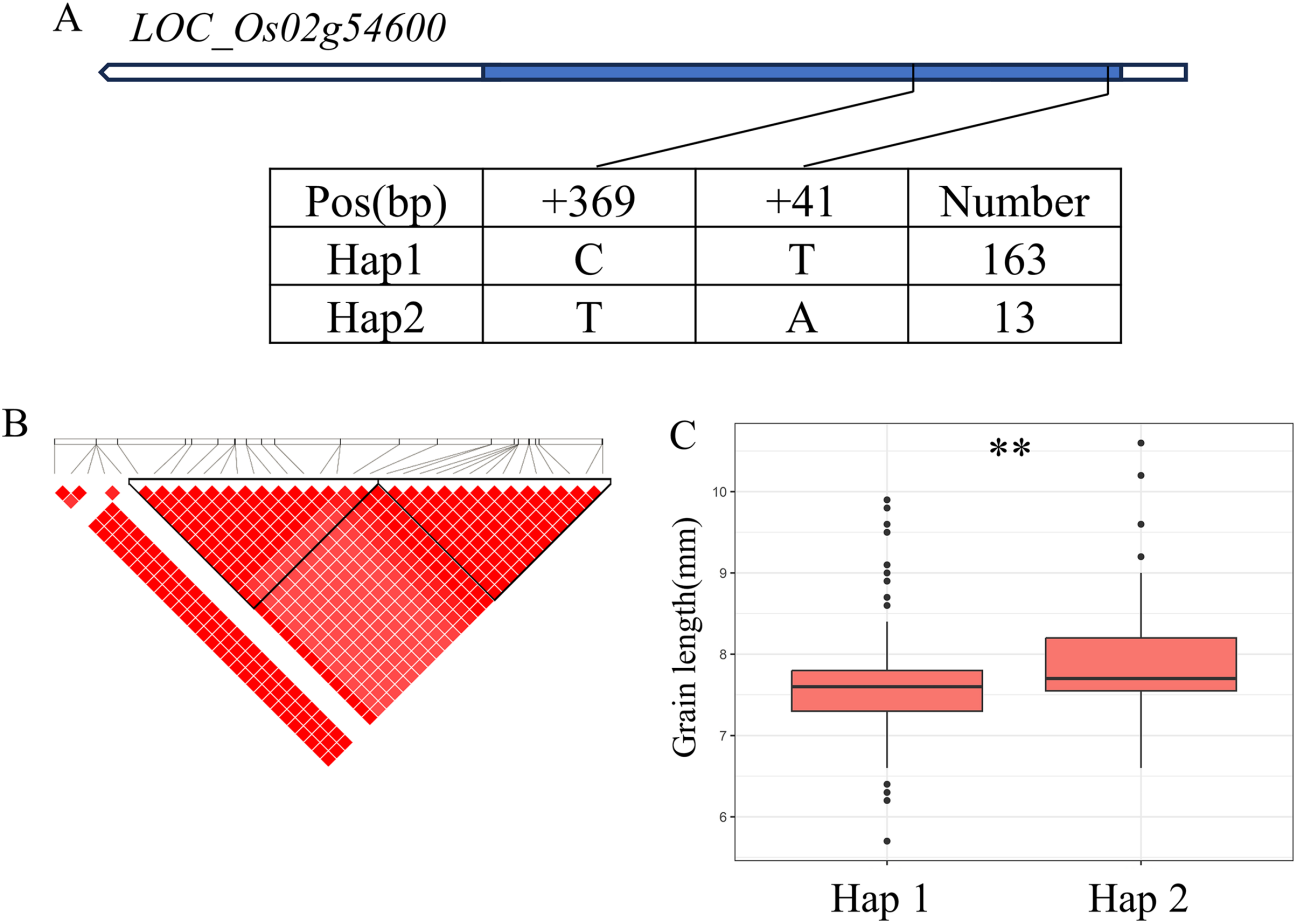
Candidate genes analysis of *qBA2* on chromosome 2. (A) Gene structure of *LOC_Os02g54600* and DNA polymorphisms within that gene. Blue boxes, white boxes and straight lines represent exons, introns and untranslated regions (UTR), respectively, and arrows represent gene directions. (B) Linkage disequilibrium (LD) block surrounding the peak on chromosome 2. (C) Boxplots illustrating grain length based on haplotypes for *LOC_Os02g54600* using non-synonymous SNPs within the coding region. **Denotes the significance of ANOVA at *P* < 0.01.

There are 69 candidate genes in the 33.04–33.50 Mb (462 kb) region of *qBA2* on chromosome 2, among which six genes have known annotations in the RAP-DB (Fig. 4B). One of these candidate genes, *LOC_Os02g54600*, is identical to *OsMMK4*, a previously reported small grain gene that regulates grain shape (Duan et al., 2014; Xu et al., 2018). The results of haplotype analysis showed that the coding region of *OsMMK4* contained 2 SNPs (Fig. 4A), forming two haplotypes, and the particles carrying Haplotype Hap1 materials were significantly higher than those carrying Hap2 materials (Fig. 4C). *OsMMK4* encodes a mitogen-activated protein kinase that influences rice grain shape by regulating cell proliferation, so we predict that this gene is a candidate for the *qBA2* region.

**Figure 5:**
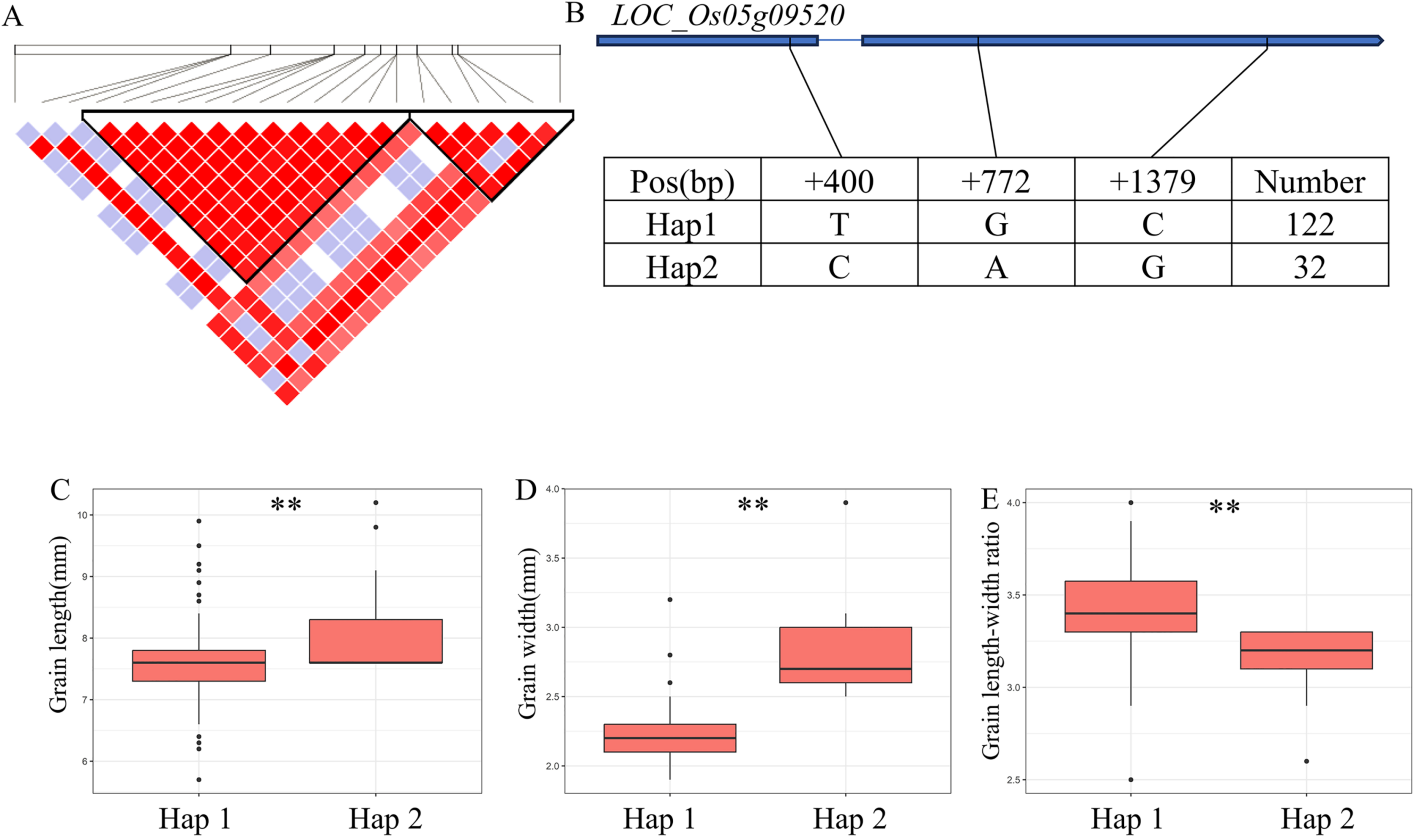
Candidate genes analysis of *qGW5* on chromosome 5. Candidate genes analysis of *qGW5* on chromosome 5. (A) Linkage disequilibrium (LD) block surrounding the peak on chromosome 5. (B) Gene structure of *LOC_Os05g09520* and DNA polymorphisms within that gene. Blue boxes, white boxes and straight lines represent exons, introns and untranslated regions (UTR), respectively, and arrows represent gene directions. (C–E) Boxplots illustrating grain length (C), grain width (D) and grain length-width ratio (E), respectively, based on haplotypes for *LOC_Os05g09520* using non-synonymous SNPs within the coding region. **Denotes the significance of ANOVA at *P* < 0.01.

For GW, BW, BR, WW and WR, a high peak of *qGW5* on chromosome 5 was mapped together with the peak of *qBW5*, *qBR5*, *qWW5*, *qWR5*. A total of 20 annotated genes were selected from the region of 5.30–5.46 Mb (161 kb) (Fig. 5A). Among them, *LOC_Os05g09520* is identical to *Grain Width on chromosome 5* (*GW5*), which regulates the expression level and growth response of brassinolide (BR) response genes (Liu et al., 2017). The coding region of *LOC_Os05g09520* contains a total of three SNPs and divides the material into two haplotypes (Fig. 5B). Hap2 showed significantly larger GL and GR values than Hap1 and showed significantly lower GW values than Hap1 (Figs. 5C–5E). Therefore, *GW5* is the most likely candidate gene in this region.

**Figure 6:**
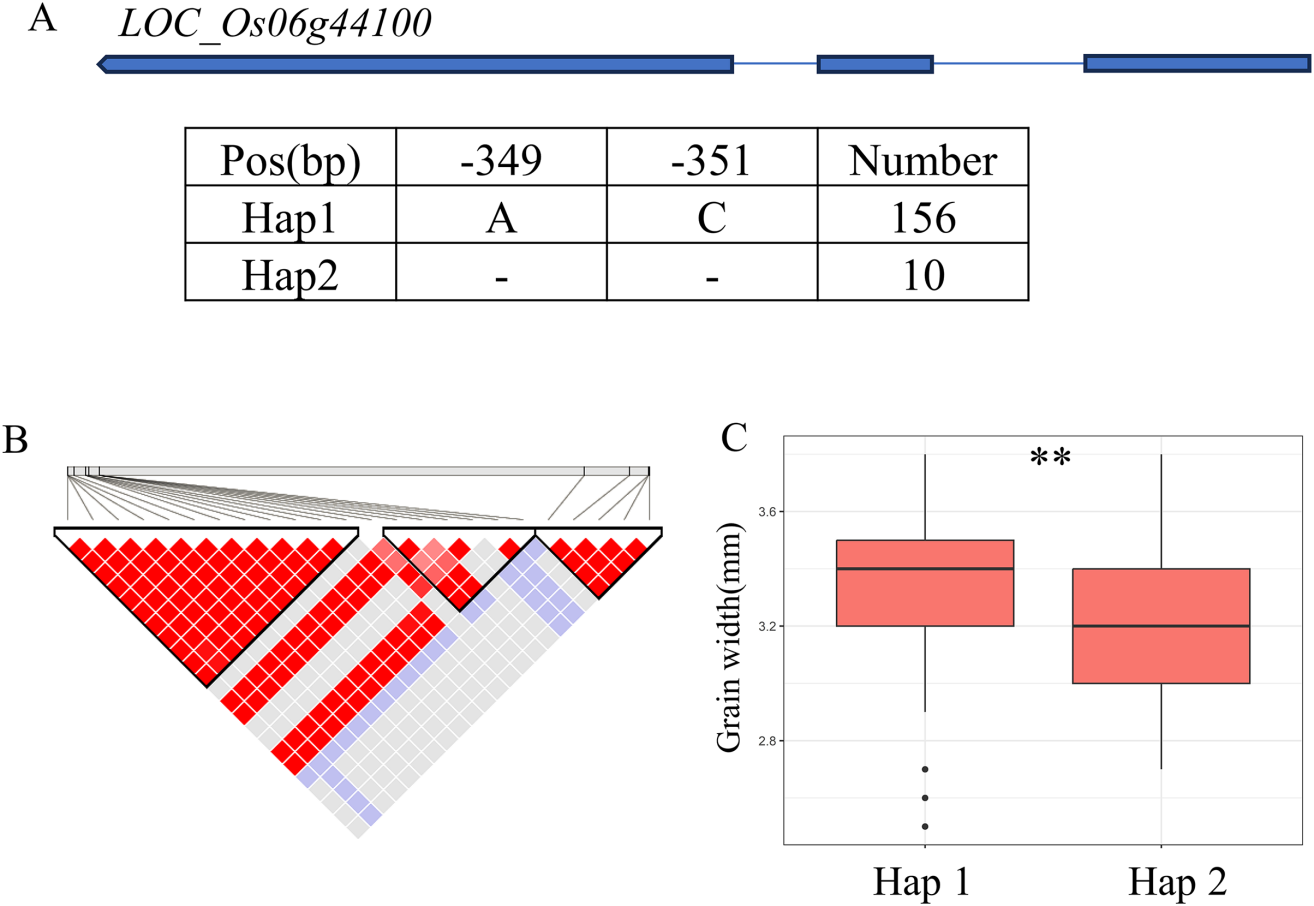
Candidate genes analysis of *qGW6* on chromosome 6. (A) Gene structure of *LOC_Os06g44100* and DNA polymorphisms within that gene. Blue boxes, white boxes and straight lines represent exons, introns and untranslated regions (UTR), respectively, and arrows represent gene directions. (B) Linkage disequilibrium (LD) block surrounding the peak on chromosome 6. (C) Boxplots illustrating grain length based on haplotypes for *LOC_Os06g44100* using SNPs within the promoter region. **Denotes the significance of ANOVA at *P* < 0.01.

A QTL cluster (*qGW6*, *qBW6*, *qBR6*, *qWW6* and *qWR6*) affecting GW was identified in the region of 26.40-26.68 Mb (279 kb) on chromosome 6, containing 40 annotated genes (Fig. 6B). Where, *LOC_Os06g44100* encoding a GNAT-like protein, that harbors intrinsic histone acetyltransferase activity (OsglHAT1) (Song et al., 2015). No SNPs were identified in the coding region of *LOC_Os06g44100*, but two SNPS were found in the promoter region, dividing all materials into two haplotypes (Fig. 6A). Hap1 exhibited a significantly higher GW value than Hap2 haplotypes in the whole population (Fig. 6C).

**Figure 7:**
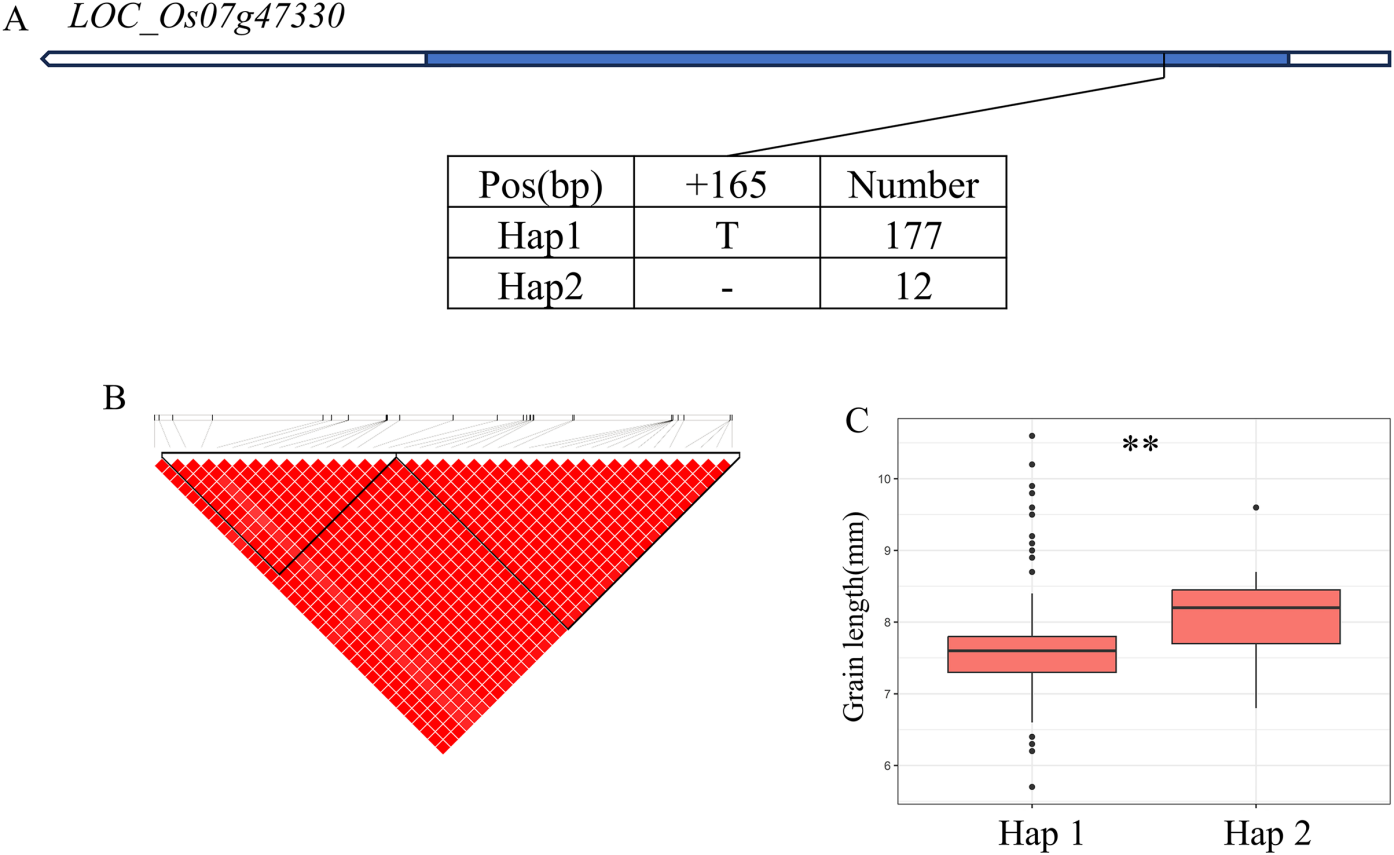
Candidate genes analysis of *qWL7* on chromosome 7. (A) Linkage disequilibrium (LD) block surrounding the peak on chromosome 7. Blue boxes, white boxes and straight lines represent exons, introns and untranslated regions (UTR), respectively, and arrows represent gene directions. (B) Gene structure of *LOC_Os07g47330* and DNA polymorphisms within that gene. (C) Boxplots illustrating grain length based on haplotypes for *LOC_Os07g47330* using non-synonymous SNPs within the coding region. **Denotes the significance of ANOVA at *P* < 0.01.

In addition, *qWL7* was identified in the region of 28.17–28.64 Mb (472 kb) on chromosome 7 (Fig. 7B), which contains 84 annotated genes. Among them, *LOC_Os07g47330* encodes a ERF domain protein, and mutations in *LOC_Os07g47330* result in small grain and dense panicle phenotypes (Komatsu et al., 2003; Bai et al., 2017; Ren et al., 2018). *LOC_Os07g47330* has only one SNP in its coding region, dividing the material into two haplotypes (Fig. 7A). The GL value of Hap1 is significantly lower than that of Hap2 (Fig. 7C).

## Discussion

Rice grain shape is a crucial agronomic trait that significantly impacts rice yield and quality. Although recent studies have identified some key regulators of grain size and molecular regulatory pathways, the understanding of the entire regulatory network remains limited and fragmented. Therefore, it is necessary to explore the upstream and downstream components of the known regulators of grain size and the possible links between the identified regulatory pathways. In this study, we conducted a genome-wide association study (GWAS) on 280 *japonica* rice varieties from northern China to decipher the genetic basis of grain shape traits. By measuring 11 grain-related traits and performing high-density single nucleotide polymorphism (SNP) genotyping, we identified 15 QTLs associated with these traits. Notably, five major QTL clusters emerged, revealing key candidate genes such as *LOC_Os01g50720*, *OsMKK4*, *GW5*, *GW6a*, and *FZP*. In comparison to previous studies, our GWAS analysis provided a more comprehensive understanding of rice grain shape by including indicators related to both brown and white rice shapes. This approach allowed us to gain insights into the interplay between glume development, grain filling, and endosperm development. For instance, we identified QTLs that specifically influence brown rice width (*qBW5*, *qBW6*) and white rice width (*qWW5*, *qWW6*), in addition to overall grain width (*qGW5*, *qGW6*). These findings contribute to a more nuanced understanding of the genetic control of rice grain shape. Our findings provide valuable insights into the genetic mechanisms underlying rice grain shape and suggest potential targets for marker-assisted selection to enhance rice quality and yield.

Recent research has revealed that rice grain shape is predominantly regulated by G-protein signaling, mitogen-activated protein kinase (MAPK) pathways, the ubiquitin-proteasome system, phytohormone signaling, and transcriptional regulators (Zuo & Li, 2014; Li, Xu & Li, 2019; Ren, Ding & Qian, 2023). Among the five grain shape-related QTLs identified in this study, four are located within the same or adjacent regions as previously reported QTLs and cloned genes influencing rice grain shape traits. For instance, we identified *GW5* as a significant candidate gene for controlling grain width and related traits in our study. *GW5* encodes a nuclear localization protein that inhibits the activity of glycogen synthase kinase GSK2, thereby regulating the expression level of brassinolide response genes and grain growth (Weng et al., 2008; Liu et al., 2017). The consistent identification of *GW5* across different studies underscores its pivotal role in determining grain width in rice. our study reinforces this notion by concurrently identifying the influence of *GW5* on grain width, brown rice width, and milled rice width, highlighting the reliability of our phenotypic data and emphasizing the pivotal role of *GW5* in regulating grain shape in japonica rice cultivars from Northern China. In addition to *GW5*, we also identified *OsMKK4* as a candidate gene for influencing multiple grain shape traits. *OsMKK4* encodes a mitogen-activated protein kinase kinase that has been previously reported to regulate grain shape by modulating brassinosteroid (BR) responses (Xu et al., 2018). Our haplotype analysis revealed two distinct haplotypes of *OsMKK4*, with Hap2 showing significantly higher grain length values compared to Hap1. This finding suggests that variations in *OsMKK4* may contribute to differences in grain shape among rice varieties, providing new avenues for rice breeding through the manipulation of *OsMKK4* alleles. Furthermore, in contrast to previous studies that did not identify different genotypes of *OsMKK4* in natural materials, we have successfully identified 13 elite germplasm lines harboring a novel haplotype of this gene. Another notable candidate gene identified in our study was *GW6a*, which is associated with grain width and related traits. Previous studies have primarily linked *GW6a* with the regulation of grain weight through changes in grain length (Song et al., 2015). However, our haplotype analysis indicated that *GW6a* may also influence grain width, which may be attributed to variations in the genetic backgrounds and environmental conditions of the materials used in different studies. Mutation of *FZP* causes smaller grains and degenerated sterile lemmas. Two haplotypes of *LOC_Os07g47330* were detected, with Hap 2 being associated with a significantly larger GL value than Hap 1 (Fig. 6C), suggesting that *LOC_Os07g47330* is a likely candidate gene for *qWL7*. However, current research on the function of the *FZP* gene primarily focuses on its role in regulating the formation of axillary bud meristems, while there is limited understanding of its molecular mechanism in regulating rice grain shape. Furthermore, we identified *LOC_Os01g50720* as a candidate gene for influencing grain length. *LOC_Os01g50720* encodes an OsMYB14 transcription factor, which belongs to the MYB transcription factor gene family in rice. Although MYB family genes have primarily been associated with responses to non-biotic stresses (Kang et al., 2022), the presence of plant hormone-related response elements in these genes suggests that *OsMYB14* may play a role in regulating grain development. Further functional validation is needed to confirm the role of *OsMYB14* in rice grain shape. Despite the similarities, our study also revealed some differences compared to previous research. One possible reason for these differences could be the genetic diversity of rice varieties used in our study. Our panel consisted of 280 *japonica* rice varieties from northern China, which may have unique genetic variations compared to other rice germplasm resources. Additionally, environmental factors such as climate, soil type, and cultural practices can also influence the expression of grain shape traits, leading to variations in QTL detection across different studies. In conclusion, our GWAS analysis of japonica rice varieties from northern China provided valuable insights into the genetic mechanisms underlying rice grain shape.

Rice yield is a complex quantitative trait controlled by multiple factors and genes, with grain shape, including grain length, grain width, grain thickness, and length-to-width ratio, being one of the crucial determinants of grain weight (Sakamoto et al., 2006). Generally, rice milling quality is negatively correlated with grain length, length-to-width ratio, and length-to-thickness ratio, while it is positively correlated with grain width, grain thickness, and width-to-thickness ratio (Meng et al., 2022). Additionally, rapid grain filling in wide-grain rice can result in a looser arrangement of starch granules, leading to the formation of chalkiness, which negatively impacts the appearance and milling quality of rice (Li et al., 2022b). Therefore, selecting new varieties of narrow-grain, high-quality rice, reducing grain width to enhance rice quality, and compensating for any loss in grain weight by increasing grain length, can improve rice quality without sacrificing yield. In this study, we identified three accessions, including LX12, LG237, and SN159, that carry low-GW haplotypes of *GW5* at *qGW5*, *qBW5*, *qBR5*, *qWW5*, and *qWR5*, as well as *GW6a* at *qGW6*, *qBW6*, *qBR6*, *qWW6*, and *qWR6*. Two accessions, YG752 and MF9, carry high-GL haplotypes of *LOC_Os01g50720* at *qGL1* and *FZP* at *qWL7*. Although these candidates are not causal genes, the SNPs in their sequences are suitable for marker-assisted selection (MAS) due to the high degree of linkage disequilibrium between them. Consequently, by converting these linked SNPs into Kompetitive Allele Specific PCR (KASP) markers, MAS can be implemented to improve grain shape, potentially enhancing rice quality through the introgression of low-GW alleles (haplotypes) of GW5 and GW6a, as well as high-GL alleles of *LOC_Os01g50720* and *FZP* into high-yielding varieties. Our findings suggest potential targets for MAS in rice breeding programs aimed at enhancing both grain quality and yield. Further functional validation of the identified candidate genes and exploration of their interactions within the genetic network will be essential for advancing our understanding of rice grain shape regulation.

## Conclusions

In conclusion, our genome-wide association study of 280 *japonica* rice varieties from northern China uncovered crucial quantitative trait loci (QTLs) influencing grain shape traits. By conducting extensive haplotype analyses and leveraging functional annotations, we identified five major QTL clusters associated with grain length, width, and area, pinpointing key candidate genes such as *LOC_Os01g50720*, *OsMKK4*, *GW5*, *GW6a*, and *FZP*. These findings not only advance our understanding of the genetic architecture underlying rice grain shape but also provide valuable genetic markers for precision breeding aimed at enhancing rice quality and yield. The implementation of marker-assisted selection strategies incorporating these loci and genes holds significant promise for improving rice varieties and contributing to global food security.

## Supplemental Information

10.7717/peerj.18746/supp-1 Supplemental Information 1 Genome-wide association results for grain-shape-related traits. Manhattan plots (left) and quantile-quantile plots (right) associated with GL, GW, GR, BL, BW, BR, WL, WW and WR in 280 accessions. For the Manhattan plots, -log10 *P*-values from a genome-wide scan were plotted against the position of the SNPs on each of 12 chromosomes and the horizontal red lines show the suggestive threshold *P* = 1.0 × 10^−4^. For the quantile-quantile plots, the horizontal axes indicate the −log10-transformed expected *P* values, and the vertical axes indicate the −log10-transformed observed *P*-values.

10.7717/peerj.18746/supp-2 Supplemental Information 2 Summary of 280 rice accessions and the grain-shape-related traits.

10.7717/peerj.18746/supp-3 Supplemental Information 3 The distribution of single nucleotide polymorphism (SNP) markers on chromosomes.

## Additional Information and Declarations

### DNA Deposition

The following information was supplied regarding the deposition of DNA sequences: The genotypes described here are available at FigShare: Chen, Hongwei (2024). Genotype of japonica accessions from northern China. figshare. Dataset. https://doi.org/10.6084/m9.figshare.26461429.v1.

### Data Availability

The following information was supplied regarding data availability: The raw measurements are available in the Supplemental File.

## References

[ref-1] Awad-Allah MMA, Mohamed AH, El-Bana MA, El-Okkiah SAF, Abdelkader MFM, Mahmoud MH, El-Diasty MZ, Said MM, Shamseldin SAM, Abdein MA (2022). Assessment of genetic variability and bran oil characters of new developed restorer lines of rice (*Oryza sativa* L.). Genes.

[ref-2] Bai X, Huang Y, Hu Y, Liu H, Zhang B, Smaczniak C, Hu G, Han Z, Xing Y (2017). Duplication of an upstream silencer of FZP increases grain yield in rice. Nature Plants.

[ref-3] Duan P, Rao Y, Zeng D, Yang Y, Xu R, Zhang B, Dong G, Qian Q, Li Y (2014). SMALL GRAIN 1, which encodes a mitogen-activated protein kinase kinase 4, influences grain size in rice. The Plant Journal: For Cell and Molecular Biology.

[ref-4] Fan C, Xing Y, Mao H, Lu T, Han B, Xu C, Li X, Zhang Q (2006). GS3, a major QTL for grain length and weight and minor QTL for grain width and thickness in rice, encodes a putative transmembrane protein. Theoretical and Applied Genetics.

[ref-5] Feng Y, Lu Q, Zhai R, Zhang M, Xu Q, Yang Y, Wang S, Yuan X, Yu H, Wang Y, Wei X (2016). Genome wide association mapping for grain shape traits in indica rice. Planta.

[ref-6] Gao X, Zhang J-Q, Zhang X, Zhou J, Jiang Z, Huang P, Tang Z, Bao Y, Cheng J, Tang H, Zhang W, Zhang H, Huang J (2019). Rice qGL3/OsPPKL1 functions with the GSK3/SHAGGY-like kinase OsGSK3 to modulate brassinosteroid signaling. The Plant Cell.

[ref-7] Hao J, Wang D, Wu Y, Huang K, Duan P, Li N, Xu R, Zeng D, Dong G, Zhang B, Zhang L, Inzé D, Qian Q, Li Y (2021). The GW2-WG1-OsbZIP47 pathway controls grain size and weight in rice. Molecular Plant.

[ref-8] Hirano K, Yoshida H, Aya K, Kawamura M, Hayashi M, Hobo T, Sato-Izawa K, Kitano H, Ueguchi-Tanaka M, Matsuoka M (2017). SMALL ORGAN SIZE 1 and SMALL ORGAN SIZE 2/DWARF AND LOW-TILLERING form a complex to integrate auxin and brassinosteroid signaling in rice. Molecular Plant.

[ref-9] Ishimaru K, Hirotsu N, Madoka Y, Murakami N, Hara N, Onodera H, Kashiwagi T, Ujiie K, Shimizu B-I, Onishi A, Miyagawa H, Katoh E (2013). Loss of function of the IAA-glucose hydrolase gene TGW6 enhances rice grain weight and increases yield. Nature Genetics.

[ref-10] Kabir MR, Nonhebel HM (2021). Reinvestigation of THOUSAND-GRAIN WEIGHT 6 grain weight genes in wheat and rice indicates a role in pollen development rather than regulation of auxin content in grains. Theoretical and Applied Genetics.

[ref-11] Kan Y, Mu X-R, Zhang H, Gao J, Shan J-X, Ye W-W, Lin H-X (2022). TT2 controls rice thermotolerance through SCT1-dependent alteration of wax biosynthesis. Nature Plants.

[ref-12] Kang HM, Sul JH, Service SK, Zaitlen NA, Kong SY, Freimer NB, Sabatti C, Eskin E (2010). Variance component model to account for sample structure in genome-wide association studies. Nature Genetics.

[ref-13] Kang L, Teng Y, Cen Q, Fang Y, Tian Q, Zhang X, Wang H, Zhang X, Xue D (2022). Genome-wide identification of R2R3-MYB transcription factor and expression analysis under abiotic stress in rice. Plants.

[ref-14] Komatsu M, Chujo A, Nagato Y, Shimamoto K, Kyozuka J (2003). FRIZZY PANICLE is required to prevent the formation of axillary meristems and to establish floral meristem identity in rice spikelets. Development.

[ref-15] Li P, Chen Y-H, Lu J, Zhang C-Q, Liu Q-Q, Li Q-F (2022a). Genes and their molecular functions determining seed structure, components, and quality of rice. Rice.

[ref-16] Li Q, Deng F, Zeng Y, Li B, He C, Zhu Y, Zhou X, Zhang Z, Wang L, Tao Y, Zhang Y, Zhou W, Cheng H, Chen Y, Lei X, Ren W (2022b). Low light stress increases chalkiness by disturbing starch synthesis and grain filling of rice. International Journal of Molecular Sciences.

[ref-17] Li Y, Fan C, Xing Y, Jiang Y, Luo L, Sun L, Shao D, Xu C, Li X, Xiao J, He Y, Zhang Q (2011). Natural variation in GS5 plays an important role in regulating grain size and yield in rice. Nature Genetics.

[ref-18] Li Z, Gui R, Yu X, Liang C, Cui J, Zhao X, Zhang X, Yu P, Chen W, Sun J (2022c). Genetic basis of the early heading of high-latitude weedy rice. Frontiers in Plant Science.

[ref-19] Li N, Xu R, Li Y (2019). Molecular networks of seed size control in plants. Annual Review of Plant Biology.

[ref-20] Li M-X, Yeung JMY, Cherny SS, Sham PC (2012). Evaluating the effective numbers of independent tests and significant p-value thresholds in commercial genotyping arrays and public imputation reference datasets. Human Genetics.

[ref-21] Lipka AE, Tian F, Wang Q, Peiffer J, Li M, Bradbury PJ, Gore MA, Buckler ES, Zhang Z (2012). GAPIT: genome association and prediction integrated tool. Bioinformatics.

[ref-22] Liu J, Chen J, Zheng X, Wu F, Lin Q, Heng Y, Tian P, Cheng Z, Yu X, Zhou K, Zhang X, Guo X, Wang J, Wang H, Wan J (2017). GW5 acts in the brassinosteroid signalling pathway to regulate grain width and weight in rice. Nature Plants.

[ref-23] Lv Y, Wang Y, Noushin J, Hu H, Chen P, Shang L, Lin H, Dong G, Hu J, Gao Z, Qian Q, Zhang Y, Guo L (2019). Genome-wide association analysis and allelic mining of grain shape-related traits in rice. Rice Science.

[ref-24] Mao H, Sun S, Yao J, Wang C, Yu S, Xu C, Li X, Zhang Q (2010). Linking differential domain functions of the GS3 protein to natural variation of grain size in rice. Proceedings of the National Academy of Sciences of the United States of America.

[ref-25] Meng B, Wang T, Luo Y, Guo Y, Xu D, Liu C, Zou J, Li L, Diao Y, Gao Z, Hu Z, Zheng X (2022). Identification and allele combination analysis of rice grain shape-related genes by genome-wide association study. International Journal of Molecular Sciences.

[ref-26] Qiao S, Sun S, Wang L, Wu Z, Li C, Li X, Wang T, Leng L, Tian W, Lu T, Wang X (2017). The RLA1/SMOS1 transcription factor functions with OsBZR1 to regulate brassinosteroid signaling and rice architecture. The Plant Cell.

[ref-100] Ren D, Ding C, Qian Q (2023). Molecular bases of rice grain size and quality for optimized productivity. Science Bulletin.

[ref-27] Ren D, Hu J, Xu Q, Cui Y, Zhang Y, Zhou T, Rao Y, Xue D, Zeng D, Zhang G, Gao Z, Zhu L, Shen L, Chen G, Guo L, Qian Q (2018). FZP determines grain size and sterile lemma fate in rice. Journal of Experimental Botany.

[ref-28] Sakamoto T, Morinaka Y, Ohnishi T, Sunohara H, Fujioka S, Ueguchi-Tanaka M, Mizutani M, Sakata K, Takatsuto S, Yoshida S, Tanaka H, Kitano H, Matsuoka M (2006). Erect leaves caused by brassinosteroid deficiency increase biomass production and grain yield in rice. Nature Biotechnology.

[ref-29] Shin J-H, Blay S, McNeney B, Graham J (2006). LDheatmap: an R function for graphical display of pairwise linkage disequilibria between single nucleotide polymorphisms. Journal of Statistical Software.

[ref-30] Si L, Chen J, Huang X, Gong H, Luo J, Hou Q, Zhou T, Lu T, Zhu J, Shangguan Y, Chen E, Gong C, Zhao Q, Jing Y, Zhao Y, Li Y, Cui L, Fan D, Lu Y, Weng Q, Wang Y, Zhan Q, Liu K, Wei X, An K, An G, Han B (2016). OsSPL13 controls grain size in cultivated rice. Nature Genetics.

[ref-31] Singh N, Singh B, Rai V, Sidhu S, Singh AK, Singh NK (2017). Evolutionary insights based on SNP haplotypes of red pericarp, grain size and starch synthase genes in wild and cultivated rice. Frontiers in Plant Science.

[ref-32] Song X-J, Huang W, Shi M, Zhu M-Z, Lin H-X (2007). A QTL for rice grain width and weight encodes a previously unknown RING-type E3 ubiquitin ligase. Nature Genetics.

[ref-33] Song XJ, Kuroha T, Ayano M, Furuta T, Nagai K, Komeda N, Segami S, Miura K, Ogawa D, Kamura T, Suzuki T, Higashiyama T, Yamasaki M, Mori H, Inukai Y, Wu J, Kitano H, Sakakibara H, Jacobsen SE, Ashikari M (2015). Rare allele of a previously unidentified histone H4 acetyltransferase enhances grain weight, yield, and plant biomass in rice. Proceedings of the National Academy of Sciences of the United States of America.

[ref-34] The 3,000 rice genomes project (2014). The 3,000 rice genomes project. GigaScience.

[ref-35] Wang S, Li S, Liu Q, Wu K, Zhang J, Wang S, Wang Y, Chen X, Zhang Y, Gao C, Wang F, Huang H, Fu X (2015a). The OsSPL16-GW7 regulatory module determines grain shape and simultaneously improves rice yield and grain quality. Nature Genetics.

[ref-36] Wang Y, Xiong G, Hu J, Jiang L, Yu H, Xu J, Fang Y, Zeng L, Xu E, Xu J, Ye W, Meng X, Liu R, Chen H, Jing Y, Wang Y, Zhu X, Li J, Qian Q (2015b). Copy number variation at the GL7 locus contributes to grain size diversity in rice. Nature Genetics.

[ref-37] Weng J, Gu S, Wan X, Gao H, Guo T, Su N, Lei C, Zhang X, Cheng Z, Guo X, Wang J, Jiang L, Zhai H, Wan J (2008). Isolation and initial characterization of GW5, a major QTL associated with rice grain width and weight. Cell Research.

[ref-38] Xia D, Zhou H, Qiu L, Jiang H, Zhang Q, Gao G, He Y (2017). Mapping and verification of grain shape QTLs based on an advanced backcross population in rice. PLOS ONE.

[ref-39] Xu R, Duan P, Yu H, Zhou Z, Zhang B, Wang R, Li J, Zhang G, Zhuang S, Lyu J, Li N, Chai T, Tian Z, Yao S, Li Y (2018). Control of grain size and weight by the OsMKKK10-OsMKK4-OsMAPK6 signaling pathway in rice. Molecular Plant.

[ref-40] Xu C, Liu Y, Li Y, Xu X, Xu C, Li X, Xiao J, Zhang Q (2015). Differential expression of GS5 regulates grain size in rice. Journal of Experimental Botany.

[ref-41] Yang X, Zhao X, Dai Z, Ma F, Miao X, Shi Z (2021). OsmiR396/growth regulating factor modulate rice grain size through direct regulation of embryo-specific miR408. Plant Physiology.

[ref-42] Yano K, Yamamoto E, Aya K, Takeuchi H, Lo P-C, Hu L, Yamasaki M, Yoshida S, Kitano H, Hirano K, Matsuoka M (2016). Genome-wide association study using whole-genome sequencing rapidly identifies new genes influencing agronomic traits in rice. Nature Genetics.

[ref-43] Zhang X, Wang J, Huang J, Lan H, Wang C, Yin C, Wu Y, Tang H, Qian Q, Li J, Zhang H (2012). Rare allele of OsPPKL1 associated with grain length causes extra-large grain and a significant yield increase in rice. Proceedings of the National Academy of Sciences of the United States of America.

[ref-44] Zhang P, Zhong K, Zhong Z, Tong H (2019). Genome-wide association study of important agronomic traits within a core collection of rice (*Oryza sativa* L.). BMC Plant Biology.

[ref-45] Zuo J, Li J (2014). Molecular genetic dissection of quantitative trait loci regulating rice grain size. Annual Review of Genetics.

